# Current Views on Infective Endocarditis: Changing Epidemiology, Improving Diagnostic Tools and Centering the Patient for Up-to-Date Management

**DOI:** 10.3390/life13020377

**Published:** 2023-01-30

**Authors:** Giovanni Cimmino, Roberta Bottino, Tiziana Formisano, Massimiliano Orlandi, Daniele Molinari, Simona Sperlongano, Pasquale Castaldo, Saverio D’Elia, Andreina Carbone, Alberto Palladino, Lavinia Forte, Francesco Coppolino, Michele Torella, Nicola Coppola

**Affiliations:** 1 Department of Translational Medical Sciences, Section of Cardiology, University of Campania Luigi Vanvitelli, 80131 Naples, Italy; 2 Cardiology Unit, Azienda Ospedaliera Universitaria Luigi Vanvitelli, 80138 Napoli, Italy; 3 Department of Women, Child and General and Specialized Surgery, Section of Anaesthesiology, University of Campania Luigi Vanvitelli, Piazza Miraglia 2, 80138 Naples, Italy; 4 Department of Translational Medical Sciences, Section of Cardiac Surgery and Heart Transplant, University of Campania Luigi Vanvitelli, 81100 Caserta, Italy; 5 Department of Mental Health and Public Medicine, Section of Infectious Diseases, University of Campania Luigi Vanvitelli, 81100 Caserta, Italy

**Keywords:** infection, antibiotics, imaging technique, multidisciplinary team

## Abstract

Infective endocarditis (IE) is a rare but potentially life-threatening disease, sometimes with longstanding sequels among surviving patients. The population at high risk of IE is represented by patients with underlying structural heart disease and/or intravascular prosthetic material. Taking into account the increasing number of intravascular and intracardiac procedures associated with device implantation, the number of patients at risk is growing too. If bacteremia develops, infected vegetation on the native/prosthetic valve or any intracardiac/intravascular device may occur as the final result of invading microorganisms/host immune system interaction. In the case of IE suspicion, all efforts must be focused on the diagnosis as IE can spread to almost any organ in the body. Unfortunately, the diagnosis of IE might be difficult and require a combination of clinical examination, microbiological assessment and echocardiographic evaluation. There is a need of novel microbiological and imaging techniques, especially in cases of blood culture-negative. In the last few years, the management of IE has changed. A multidisciplinary care team, including experts in infectious diseases, cardiology and cardiac surgery, namely, the Endocarditis Team, is highly recommended by the current guidelines.

## 1. Introduction

Infective endocarditis (IE) is a rare disease involving the endocardial surface and mainly the heart valves. However, it should be considered a systemic disease, involving multiple organs such as the brain, lung, vertebral column and spleen, and the embolic phenomena might be the symptoms of the disease at presentation. Despite the availability of broad-spectrum antibiotics and the diffusion of more accurate diagnostic techniques, such as transoesophageal echocardiography and positron emission tomography, the mortality rate of IE still remains high at up to 18–25% in the first 3 months [1].

In the 21st century, the IE scenario has changed because of various aspects: first, rheumatic fever has declined, thus becoming a less frequent predisposing factor; and second, a growing number of IE affects elderly patients with cardiac devices or transcatheter heart valves. Indeed, the percutaneous approach opened up the possibility of treating some valvular diseases such as aortic stenosis in patients with a prohibitive surgical risk but with a not-negligible increase in IE with a poor prognosis [2,3]. Considering the complexity of the management and high mortality of IE, it has been proposed to set up, inside the referral hospital for IE, an “Endocarditis Team” composed of multiple figures (cardiologists, infective disease specialists, heart surgeons and radiologists) in order to optimize the management and improve the prognosis of IE [4,5]. Some recent studies demonstrated that a multidisciplinary approach shortened the time to diagnosis and improved the survival of patients with IE [6,7]. IE has a great economic impact. The standard treatment for IE is based on 4 to 6 weeks of intravenous (IV) antibiotic therapy, thus the cost for its management has to take into account the long hospitalization and cost of the IV therapy [8]

In this review, we will summarize the emerging aspects of IE in terms of changing epidemiology and microbiology, a novel diagnostic technique and evolving management with a look to the aspects that should be improved in the prevention, diagnosis and treatment of this disease. Moreover, taking into account that in the last few decades there has been an increase in cardiac devices implantation, we will also analyze cardiac device-related infective endocarditis.

## 2. Epidemiological Impact of an Old Disease: Emerging Risk Factors?

The incidence of IE is estimated to be around 3–7/100,000 person-years, which has apparently been increasing over the last decades [4]. The in-hospital mortality rate is high, ranging from 14 to 22% [9,10], with a high incidence of per year mortality (up to 15–30%) [4] and a five-year survival rate, similar or even worse, than some cancers [4,11]. Even if the incidence is higher in men (with a male/female ratio ≥ 2:1), females have a worse prognosis and apparently undergo valve repair less frequently [12,13]. The increasing incidence of IE in the last 30 years, in the absence of improvement in outcomes and mortality, makes it a disease with a high impact on public health resources in terms of hospitalization and treatment costs [14]. Classically, the patients considered at higher risk of developing IE are the carriers of prosthetic valves or prosthetic material used in valve repairs, patients with cyanotic congenital heart disease who have not undergone cardiac surgery or with incomplete repair of intracardiac shunts, and patients with a previous history of IE [4]. Antibiotic prophylaxis is recommended for these patients when awaiting dental procedures [4].

Rheumatic and congenital valvular heart diseases have been the most important risk factors for endocarditis development, especially in young patients [15]. In developed countries, the epidemiology of IE has undergone significant changes, with emerging risk factors such as the presence of intracardiac devices (defibrillators and pacemakers mainly) and degenerative valvular disease, thus shifting the incidence of IE towards an older age (≥65 years old) [16,17].

Moreover, the last opioid epidemic has considerably affected the current epidemiology of IE in developed countries, especially in the USA, where there is an increased incidence of tricuspid valve endocarditis due to injection drug use, mainly affecting women and young people [18,19,20,21].

Lastly, other well-recognized risk factors for IE are poor dentition or dental procedure, immunosuppression and hemodialysis [22].

## 3. Microbiological Burden of Infective Endocarditis: A Changing Scenario?

In the last two decades, the population at risk has changed, thus contributing to a shift in the microbiology of IE too [23]. Indeed, whereas subacute IE caused by streptococcus viridans was once the most common [24], acute staphylococcus aureus infections now appear to be predominant, being frequently encountered in injection drug use [25] and device-related IE [26].

Especially in the latter, the infection is often caused by multidrug-resistant *S. aureus* (methicillin-resistant, MRSA) turning antibiotic therapy into a challenge in this patient population [27,28].

The third most common group of gram-positive bacteria responsible for IE is the group of enterococci, normally associated with community-acquired infections or urinary or gastrointestinal abnormalities (enterococcus faecalis) [29]. Interestingly, the incidence of enterococci-related nosocomial endocarditis seems to be increasing, particularly in patients undergoing TAVI [3,30], possibly in relationship to the standard population undergoing the intervention (older age even in native valves) [31] and the femoral puncture required for the procedure [32]. This evidence raises questions about the most appropriate antibiotic prophylaxis to be administered in patients undergoing TAVI (cephalosporins vs. amoxicillin/clavulanic acid) [33]. Bacterial species of staphylococci, streptococci and enterococci alone are estimated to account for approximately 70–80% of all cases of endocarditis: staphylococci (40–45%, mainly *S. aureus*), streptococci (35–40%, mainly viridans streptococci) and enterococci (10%) [34,35].

**Figure 1:**
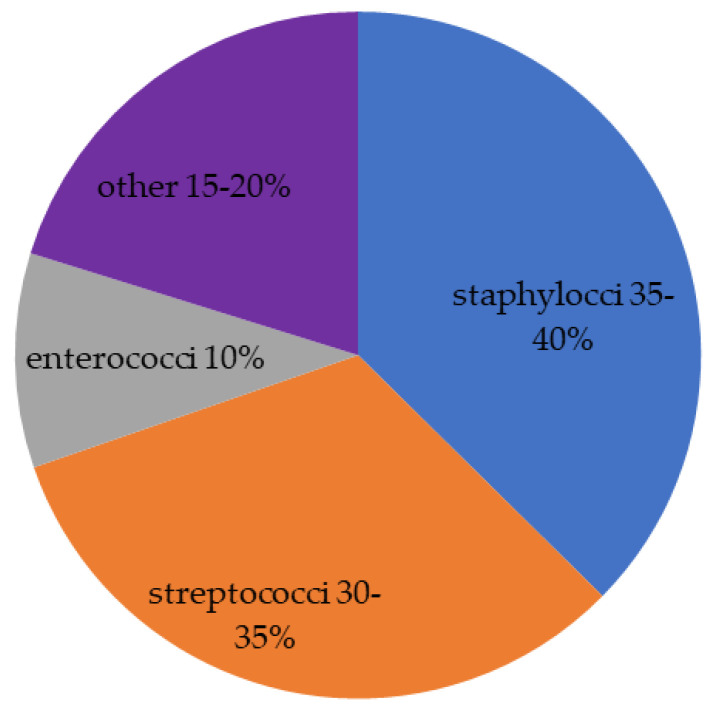
Distribution of pathogens mainly involved in IE. The last group includes HACEK group (2%), fungi, polymicrobial infection and, rarely, aerobic Gram-negative bacilli.

**Figure 2:**
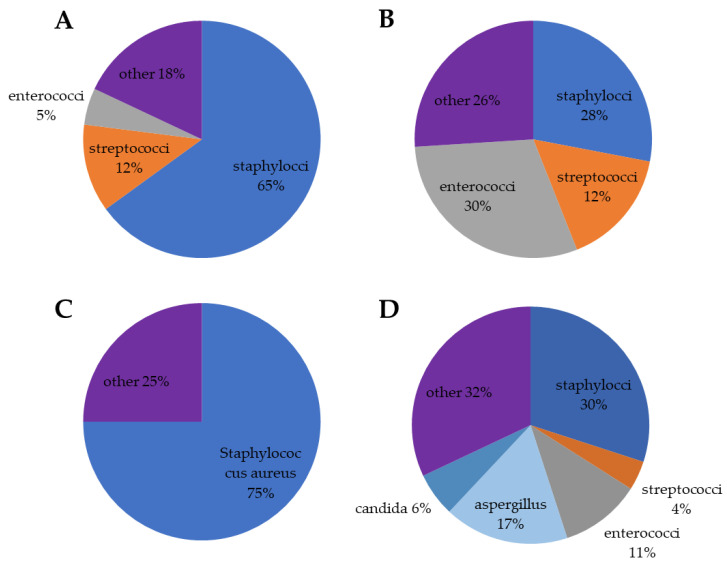
Distribution of pathogens in different clinical settings. (**A**) cardiac device related-infective endocarditis; the other group includes *Candida* spp., other fungi, Gram-negative bacilli and polymicrobial. (**B**) transcatheter aortic valve implantation (TAVI); the other group includes Gram-negative bacille, Moraxella, candida albicans, Histoplasma and corynebacterium. (**C**) right-sided infective endocarditis; the other group includes streptococci, coagulase-negative staphylococcal, pseudomonas aeruginosa and fungi. (**D**) immunosuppressive therapy in solid organ transplantation; the other group includes Gram-negative bacilli, Corynebacteria, Clostridium ramosum, Pseudallescheria boydii, Nocardia asteroids and Polymicrobial.

Concluding this point, *Enterococci* spp. shows several therapeutical problems: cephalosporins are not active against Enterococci; penicillins have a bacteriostatic effect; the prevalence of ampicillin and vancomicyn-resistance is considerable for *E. foecalis* and very high and high, respectively, for *E. foecium*. The HACEK group (haemophilus species, aggregatibacter species, cardiobacterium species, Eikenella corrodens and kingella species), fungi, polymicrobial infection and, rarely, aerobic Gram-negative bacilli are isolated in 5–10% of cases [36]. The HACEK (haemophilus species, aggregatibacter species, cardiobacterium species, Eikenella corrodens and kingella species) are Gram-negative bacteria and difficult to isolate in culture. They correspond to 1.8% of the total cases of endocarditis, with a higher prevalence in patients with prosthetic valves [36]. Fungal endocarditis is rare for both native valves and cardiac devices and is most associated with cardiovascular implantable electronic device-related infective endocarditis (CIED-IE) and prosthetic valve endocarditis (PVE) [37]. Candida and Aspergillus species are the main causative fungal microbes. Fungal endocarditis is extremely difficult to diagnose, and the initiation of appropriate treatment is often delayed (82% receiving a late or incorrect diagnosis, 28–77% only a post-mortem diagnosis). The mortality rate is very high (72%) [38,39]. In Figure 1 is reported the distribution of the different pathogens isolated in all cases of IE. However, the prevalence of microorganisms varies considerably in relation to the specific clinical setting as reported in Figure 2.

### 3.1. Cardiac Device Related-Infective Endocarditis

CIED-IE are characterized by a significant prevalence of staphylococcal infections, accounting for up to 65%. Compared to the past, there is a decline in coagulase-negative staphylococcus infections (27%), with the emergence of *S. Aureus* as the primary cause of CIED-IE (38%). The frequent infectious agents are other gram-positive cocci at 21% (mainly streptococci 12% and enterococci 5%) [40,41].

### 3.2. Prosthetic Valve Endocarditis

Staphylococci (32%), streptococci (25%) and enterococci (16%) are the main causes of prosthetic valve endocarditis (PVE). Transcatheter aortic valve implantation (TAVI) deserves a separate mention as the main isolated infectious agent is *Enterococcus* spp. (25–30%), especially for early or peri-procedural infections (30–35%) [2,42,43,44].

### 3.3. Right-Sided Infective Endocarditis

Right-sided IE accounts for 5 to 10% of all endocarditis cases. Compared with left-sided IE, it is more frequently associated with intravenous drug use, intracardiac devices and central venous catheters. Staphylococcus aureus is the predominant infectious agent (60–90% of cases). Streptococcal and coagulase-negative staphylococcal infections are also frequent causes of right-sided IE. A higher prevalence of Pseudomonas aeruginosa and other Gram-negative bacteria is described in right-sided IE [45].

### 3.4. Immunosuppressive Therapy in Solid Organ Transplantation

The spectrum of organisms causing IE is quite different in transplant recipients compared to the general population. Fifty percent of the infections were due to Aspergillus fumigatus (17%) or Staphylococcus aureus (30%), while the prevalence of viridans streptococci infections was lower (4%) than IE in immunocompetent patients. The Enterococcus species (11%) and Candida species (6%) represent other common agents. Fungal infections accounted for the most frequent etiologic agent within 30 days of transplantation (60%), while bacterial infections caused the majority of cases (80%) after this period [46,47,48,49].

## 4. Diagnosis of Endocarditis: New Techniques on the Way?

The diagnosis of IE is based on clinical manifestations, the identification of the infective pathogen by blood cultures or other microbiologic tests and the detection of valvular vegetations or the infection’s structural complications using cardiac imaging. The accepted criteria for IE’s diagnosis are the 2000 modified Duke’s criteria, which classify IE as definite, possible or rejected [50,51]. The modified Duke’s criteria were developed for the evaluation of patients with left-sided native valve IE, and their diagnostic accuracy is lower in patients with suspected right-sided native valve IE, prosthetic valve IE and pacemaker or defibrillator lead IE, for which echocardiography can be normal or inconclusive [52]. The sensitivity of the Duke’s criteria can be improved by other imaging modalities including MR, CT, PET/CT and SPECT/CT, aiming at evaluating cardiac involvement and embolic events. The choice of a particular diagnostic modality will be guided by local availability and expertise [53].

Electrocardiography and chest radiography should always be performed as part of the initial evaluation of patients with suspected IE. The presence of heart block or conduction delay at baseline electrocardiography may be indicative of paravalvular extension of the infection. Moreover, the finding of myocardial ischemia may suggest the occurrence of septic emboli in the coronary arteries. Chest radiography may show the presence of septic pulmonary emboli or potential alternative causes of fever and systemic symptoms [53].

### 4.1. Left-Sided Native Valve Endocarditis

IE of the left-sided native valve should be suspected in patients with relevant cardiac risk factors (e.g., pre-existing valvular or congenital heart disease), prior IE and other predisposing conditions, including intravenous drug use, immunosuppression, recent dental or surgical procedure, an indwelling cardiac device or intravenous catheter. Positive blood cultures, clinical features and echocardiographic findings remain the cornerstones for left-sided native valve IE’s diagnosis. At least three sets of blood cultures should be obtained at 30 min intervals from separate venipuncture sites before starting antibiotic therapy [54,55]. Each set consists of one aerobic and one anaerobic bottle, each of which contains a volume of blood of 10 mL. Samples from peripheral veins using a meticulous sterile technique are preferred over central venous catheter due to the risk of contamination. As bacteremia is usually continuous in patients affected by IE, blood cultures can be collected at any time and not necessarily when fever or chills occur. Blood culture results should be interpreted according to the modified Duke’s criteria. Most clinically significant bacteremias are usually detected within 48 h; the pathogen members of the HACEK group can be identified after 5 days of incubation with modern detection systems [36]. Occasionally, false-positive blood cultures can occur, due to the presence of contaminants. Contamination likelihood is reduced when the microorganism is found in multiple blood cultures obtained from different venipuncture sites [56]. In patients with negative blood cultures after 5 days of incubation and subculturing using usual methods, blood culture-negative IE should be suspected when persistent fever, signs of embolism or vegetations on echocardiography are observed [57]. Culture-negative IE may be caused by previous antibiotic therapy or fungi or fastidious intracellular bacteria, which require specialized culture media and grow relatively slowly [58]. In these cases, other microbiologic tests, such as serology or PCR, may be needed to identify the responsible pathogens (e.g., *Coxiella burnetii*, *Tropheryma whipplei*, *Bartonella* spp., *Aspergillus* spp., *Candida* spp., *Chlamydia* spp., *Legionella* spp., *Mycoplasma pneumoniae* and *Brucella* spp.) [58], as reported in Table 1.

Finally, the culture of resected heart valves may be useful when blood cultures are negative, even if it is not recommended in the absence of clinical suspicion because of the risk of false-positive findings [59].

Echocardiography, either TTE or TOE, is the mainstay of cardiac imaging for IE diagnosis, and it must be performed as soon as IE is suspected [60]. It is considered positive for IE in the presence of vegetations and/or structural complications, including abscesses, leaflet perforation, aortic pseudoaneurysm and intracardiac fistula. Echocardiography is also useful to evaluate any associated mitral or aortic valve dysfunction and the underlying left ventricular function. Generally, TTE is the first diagnostic tool in patients with suspected IE, with a high specificity (close to 100%) and modest sensitivity (about 75%) [60]. In particular, false-negatives may be found if vegetations are small or have embolized. Therefore, the absence of vegetations on TTE does not exclude IE diagnosis, although the finding of a valve with normal morphology and function greatly reduces its probability [61]. TOE sensitivity is higher than TTE in detecting both vegetations and cardiac complications; thus, in most cases, TOE follows TTE in the diagnostic workup of IE [62,63]. TOE becomes necessary when TTE is negative (or the transthoracic window is poor) but the clinical suspicion of IE is high, a valve vegetation is found with concern of intracardiac complications (e.g., new conduction delay due to paravalvular abscess), and vegetation is associated with significant valvular regurgitation to be quantified before surgery. Conventional TOE may be completed by 3D analysis, which provides a more precise estimate of the vegetation’s size with a better prediction of the embolic risk [64]. If initial TOE is negative but the clinical suspicion of IE remains high, TTE and/or TOE should be repeated in 7 days, or even earlier in the case of Staphylococcus aureus infection [65,66]. False-positive findings may occur when performing TOE in the presence of cardiac tumors (e.g., valve fibroelastomas), mural thrombi or fibrous valvular strands (including Lambl’s excrescences) [67].

Therefore, ultrasound examination should always be interpreted with caution, taking into account the clinical presentation and likelihood of IE.

Cardiac CT may be considered in patients with suspicion of infective endocarditis when ultrasound examinations are non-diagnostic. Although it is inferior to TOE in detecting vegetations, cardiac CT is superior for the evaluation of the paravalvular extension of infection [68,69]. Moreover, it can be more informative than TOE when TOE is limited by artifacts [68,70,71,72]. In aortic valve IE, CT may be used to better characterize the morphology, size and calcification of aortic valve, aortic root and ascending aorta in anticipation of surgery.

MR has a higher sensitivity than CT in detecting the cerebral consequences of IE, which are most often ischemic lesions [73,74]. In particular, MR allows for a better lesion characterization in patients with IE and neurologic symptoms, whereas it plays a key diagnostic role in patients with non-definite IE and no neurologic symptoms, in whom cerebral lesions are found in at least half of the cases [75]. CT can be a more feasible and practical alternative to cerebral MR in critically ill patients with IE.

### 4.2. Right-Sided Endocarditis

Right-sided IE should be suspected in the presence of injection drug use, CIED (in which infection usually spreads from the pocket to the insertion leads), other intravascular devices, such as a central line, intra-aortic balloon pump or ventricular assist device, and underlying right-sided cardiac anomaly. Modified Duke’s criteria diagnostic sensibility for right-sided/CIED-IE is lower, as audible murmurs, peripheral emboli and immunologic and vascular phenomena are usually absent [52]. However, intravenous drug use and septic pulmonary emboli, which fall within the minor criteria, are important clues for right-sided IE.

**Figure 3:**
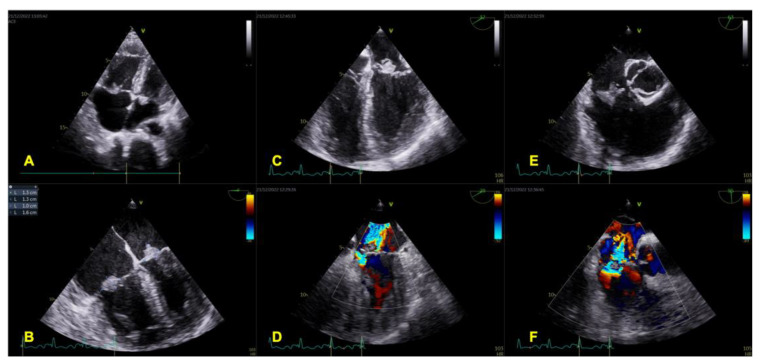
IE of both tricuspid and mitral valve. Vegetations are well-imaged by TTE, which is the first imaging tool in diagnosing IE (**A**). Their presence, size and location are confirmed by TOE (**B**,**C**,**E**). Both vegetations are associated with significant valve regurgitations (**D**,**F**). IE: infective endocarditis; TTE: transthoracic echocardiography; TOE: transesophageal echocardiography.

Among people who inject drugs, the diagnostic accuracy of TTE is comparable to TOE [76,77,78]. However, given that intravenous drug abusers are at risk of left-sided as well as right-sided IE, the diagnostic approach to echocardiography remains the same for both. Among patients with CIED infection, TOE is more sensitive than TTE in detecting lead and valve vegetations [79,80,81,82,83,84]. When intracardiac infection is highly suspected and TOE is negative or non-diagnostic, alternative testing is necessary. In particular, radiolabeled white blood cells SPECT/CT may be used for the detection, localization and extension of CIED infection [85]. Likewise, 18F-FDG PET/CT showed high diagnostic accuracy in identifying pocket/generator infections and lead-associated endocarditis [86,87,88,89,90]. Moreover, 18F-FDG PET/CT can detect septic pulmonary emboli as a manifestation of lead-related IE. Finally, CT may reveal concomitant pulmonary complications, including abscesses and infarcts. An echocardiographic evaluation of right-sided endocarditis is reported in Figure 3.

### 4.3. Prosthetic Valve Endocarditis

The diagnosis of prosthetic valve IE should be suspected in patients with history of valve replacement and positive blood cultures and/or evocative symptoms and/or new prosthetic valve dysfunction, particularly paravalvular regurgitation.

TTE is often the initial imaging test. However, TOE has higher sensitivity than TEE for detection of both the vegetation and paravalvular extension of infection, including abscess, fistula, leaflet perforation, pseudoaneurysm and paraprosthetic leak. Therefore, TOE should always be performed in patients with prosthetic valves and suspicion of IE. Even when a complete echocardiographic evaluation is performed, modified Duke’s criteria sensitivity remains lower for prosthetic than native valves. Therefore, when IE diagnosis is not definitely based on the modified Duke’s criteria but the clinical suspicion is strong, echocardiography should be repeated after 5–7 days. If the diagnosis is still uncertain, further cardiac imaging should be considered. CT angiography may be superior to TEE for the detection of paravalvular complications, including abscesses, pseudoaneurysms, fistulas and dehiscence and for planning a surgical strategy in this subset of patients [69,91,92,93]. Moreover, CT angiography may provide a satisfactory coronary imaging for patients at an intermediate risk of coronary artery disease who require surgical intervention. 18F-FDG PET/CT increases Duke’s criteria diagnostic sensitivity for prosthetic valve IE, with little loss in specificity [88,94,95]. Its results have to be interpreted with caution in patients who have recently undergone cardiac surgery, as the postoperative inflammatory response may result in non-specific 18F-FDG uptake. The use of CT angiography and/or 18F-FDG PET TC may be especially beneficial for patients with TAVI and suspected IE, where the sensitivity of echocardiography (including TOE) is limited due to artifacts and shadowing caused by the metal stent anchoring the valve [96,97]. A comparison between the different technique is provided in Table 2.

**Figure 4:**
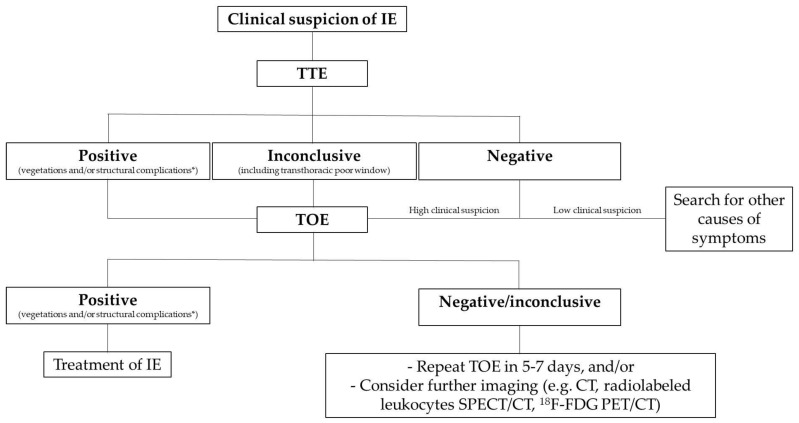
Diagnostic imaging tools in suspected IE. Structural complications include abscess, pseudoaneurysm, intracardiac fistula, valvular perforation or aneurysm and new dehiscence of prosthetic valve. CT: computed tomography; FDG: fluorodeoxyglucose; IE: infective endocarditis; PET: positron emission tomography; SPECT: single photon emission computed tomography; TOE: transesophageal echocardiography; TTE: transthoracic echocardiography.

A summary of the diagnostic flow chart is summarized in Figure 4.

## 5. Infective Endocarditis Treatment: A Gray Scale of Evidence

The mainstay of endocarditis treatment is antibiotic therapy. Surgery is equally important in the case of complications.

### 5.1. Antibiotic Therapy: General Principles

Bactericidal regimens are more effective than bacteriostatic therapy as host defenses are of little help [98,99]. The association of aminoglycosides (inhibitor of proteic synthesis) with cell-wall inhibitors is no longer recommended in staphylococcal native valve endocarditis (NVE) because of its undemonstrated clinical benefits and increased renal toxicity [100]. Aminoglycosides are still recommended for harder infections (i.e., staphylococcal PVE), given in a single daily dose to reduce nephrotoxicity [101], even if a recent meta-analysis highlights the paucity of clinical data to support this treatment regimen and suggests a downgrade of the indication due to the lack of benefit and the demonstrated nephron- and hepato-toxicity of adjunctive gentamicin or rifampin in staphylococcal PVE [102]. Treatment of NVE should last 2–6 weeks. PVE needs prolonged therapy (6 weeks) due to the antibiotic tolerance of the bacteria present in vegetation and biofilms. Bactericidal drug combinations are preferred to monotherapy against tolerant organisms. In NVE that needs a valve replacement during antibiotic therapy, the postoperative antibiotic regimen should be the same one recommended for NVE and not for PVE. Rifampin should be used only in foreign body infections, such as PVE, after 3–5 days of effective antibiotic therapy once the bacteremia has been cleared. In HACEK-related species, third-generation cephalosporins and quinolones are suggested as some HACEK group bacilli are beta-lactamases producers [103].

For non-HACEK-related endocarditis, early surgery and long-term (at least 6 weeks) therapy with bactericidal agents (betalactams plus aminoglycosides) is recommended, sometimes with the addition of quinolones or cotrimoxazole.

### 5.2. Empirical Therapy

Empirical therapy should be started as soon as possible after collecting at least three sets of blood cultures. The choice of the antibiotics depends on the kind of valve affected (native or prosthetic), the time from surgery in the case of prosthetic valves (early or late), the place of infection (community, nosocomial or non-nosocomial healthcare-associated IE) and, in the case of non-community IE, the local prevalence of multidrug-resistant microorganisms. NVE and late PVE regimens should cover staphylococci, streptococci and enterococci. Early PVE or healthcare-associated IE regimens should cover methicillin-resistant staphylococci, enterococci and, ideally, non-HACEK Gram-negative pathogens. When the pathogen is known, the therapy will be based on the antibiogram.

It is not a purpose of this article to specify each antibiotic used for IE. Here, we provide general protocols. Detailed information is available on the current guidelines [4].

In community-acquired native valves or late prosthetic valve (>12 months from surgery) endocarditis, a regimen with ampicillin + flucloxacilli or oxacillin + gentmicin is recommended. In penicillin allergic patients the combination of ampicilin + flucoxacillin/oxacillin can be replaced by vancomycin (30–60 mg/kg/day i.v. in 2–3 doses).

In early PVE (<12 months from surgery), either nosocomial or non-nosocomial healthcare-associated endocarditis, vancomycin + gentamicin + rifampin (only for PVE, 3–5 days later than vancomycin and gentamicin) is indicated.

In blood culture–negative IE (BCN-IE), no causative microorganism can be grown in the usual blood cultures. This could happen as a consequence of previous antibiotic administration, infection by fungi or obligatory intracellular. Isolation of these microorganisms requires culturing them on specialized media [58].

Most frequently, micotic IE affects prosthetic valves, i.v. drug abusers and immunocompromised patients [104]. The most common agents are Candida and *Aspergillus* spp. Due to its high mortality, therapy is based on antifungal administration and surgical valve replacement [104].

### 5.3. Cardiac Device-Related IE

Cardiac device-related IE (CDR-IE) is an infection that involves electrode leads, cardiac valve leaflets or the endocardial surface. It is distinct from local device infection which is limited to the pocket of the cardiac device [105]. CDR-IE must be treated using prolonged antibiotic therapy and complete hardware removal, preferably by transvenous lead extraction [105]. Most CDR-IE infections are secondary to staphylococcal species (often methicillin-resistant) [106] so empirical therapy should include vancomycin. Daptomycin, approved for right-side IE and bacteremia attributable to *S. aureus* [107], is a valid molecule to treat CDR-IE [108]. The therapy is indicated for 4–6 weeks in most cases [109]. After extraction of an infected device, at least 2 weeks of parenteral therapy are recommended for patients with bloodstream infections. If sustained positive blood cultures (>24 h) remain despite CIED removal and appropriate antimicrobial therapy, parenteral therapy has to be continued for at least 4 weeks [105,109]. If indication to implant persists, the new device should be reimplanted on the contralateral side [105,110]. Not a clear recommendation concerning the optimal timing of reimplantation exists to date. Immediate reimplantation should be avoided because of the risk of new infection. Negative blood cultures for at least 72 h before reimplantation are highly recommended. In cases of remnant valvular infection, implantation should be delayed for at least 2 weeks. Temporary pacing is a risk factor for subsequent CDR-IE, venous thrombosis and a prolonged hospital stay because the patient has to remain on telemetry and bed rest due to the risk of displacement and failure to capture, thus it should be avoided if possible [105,111]. Several studies have evaluated the safety and efficacy of surgical epicardial pacemaker implantation at the moment of lead extraction [112]. A low risk of re-infection has been reported, concluding that an epicardial pacemaker might be used as a bridge until a permanent intravenous CIED is implanted or as a replacement for a permeant intravenous CIED [113,114,115].

### 5.4. Surgical Treatment

Surgery is necessary in up to 50% of patients with IE [116,117]. Most patients do not undergo surgery because the prohibitive surgical risk due to comorbidities, age or IE complications or, in a minor percentage of cases, the infection heals with medical therapy alone [118]. The ESC/AHA/AACT guidelines give recommendations on surgery with a level of evidence B (or C) [4,65,119], given the lack of evidence due to the ethical issue of patient selection in randomized trials. The aim of a surgical procedure is the removal of the infected or damaged valve tissue, the cleansing of any abscesses and the repair of a fistula or false aneurysm. If possible, the valve is spared; otherwise, it is replaced with a biological or mechanical prosthesis. Surgery is indicated in all cases in which antibiotic therapy alone is not sufficient to improve the patient’s prognosis. The three main indications to surgery are treatment of heart failure caused by valve regurgitation, difficult to control heart infection and recurrence of embolism. For each patient, the decision should be made by the endocarditis team.

A new promising system for the treatment of both tricuspid valve and CIED IE is the Angio-Vac system, a percutaneous system of aspiration approved in 2014 by the FDA for thrombi aspiration in pulmonary embolism. This system is useful for right-side IE (tricuspid valve and CIED) when there is not complete valve destruction and for patients considered at high risk for surgery [112,120]. In an observational study of 33 patients [121], Angio-Vac is used to remove large vegetation (dimension 2.0 ± 0.7 cm) with a successful rate of 61%. For CIED infection, this system is used to remove the infected material adhering to the leads reducing the risk of embolization during the extraction [120,121]. However, the Angio-Vac system for the treatment of IE is still experimental, and further studies comparing it with medical therapy or surgery will be needed so that the next guidelines can include it in the recommendations.

## 6. Prophylaxis

Prophylaxis is recommended in patients considered at a high risk of IE [122], who undergo dental procedures requiring manipulation of the gingival or periapical region of the teeth or perforation of the oral mucosa. Patients considered at the highest risk of IE are patients with a prosthetic valve/prosthetic material used for valve repair [123], patients with previous IE [124] and patients with untreated cyanotic congenital heart disease and those with CHD who have postoperative palliative shunts, conduits or other prostheses [125,126]. After surgical repair with no residual defects, LGs recommend prophylaxis for the first 6 months after the procedure [125,127].

## 7. The Endocarditis Team: A Multi-View for One Disease

The current literature supports the benefits of the multidisciplinary management of IE [128,129,130], which results in a significative reduction in the overall in-hospital mortality and 1-year survival. For this reason, current guidelines recommend that a specialized team (Endocarditis Team) is institutionalized in reference centers for the management of patients with IE [4,5,65,130].

**Figure 5:**
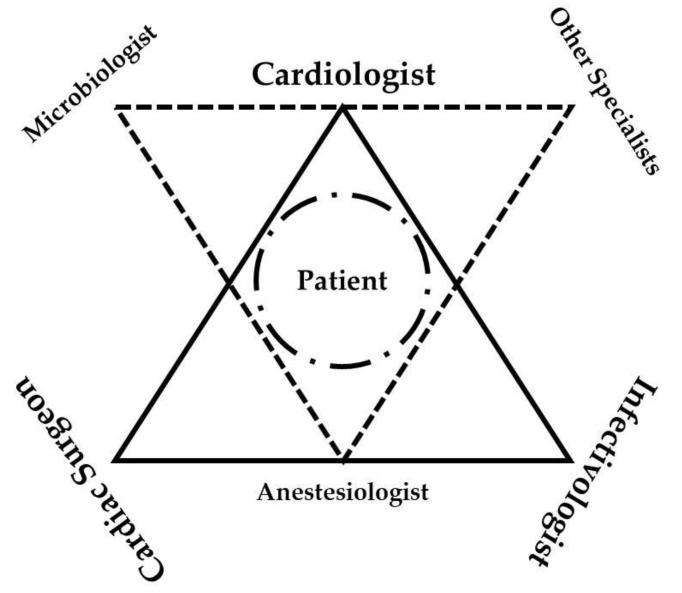
Schematic view of Endocarditis Team. The continuous line indicates the main specialists involved in the decision-making process where the patient is in the center. Discontinuous line indicates the other medical specialists that might be involved according to the single case.

In our center, the Endocarditis Team has recently brought together the expertise of different specialists, such as cardiologists (experienced to perform transthoracic and transesophageal echocardiogram), microbiologists, infectious disease specialists, cardiac surgeons, radiologists and other physicians that are involved according to the clinical status of the patients (neurologists, oncologists and generic or vascular surgeons), as summarized in Figure 5. It is an advancement in the management of IE centering any therapeutic decision on the medical background of each patient.

## 8. Conclusions

Infective endocarditis is an evolving disease also because of the growth in mini-invasive cardiac procedures in the last few years. An up-to-date management should require (1) a preventive strategy to reduce the burden of morbidity and mortality especially in high risk patients; (2) improved diagnoses with early echocardiographic evaluation, rapid microbiological results and advanced imaging techniques when required; and (3) optimal therapeutical strategies based on the Endocarditis Team’s discussion, early risk stratification, tailored antibiotic therapy, early surgery if indicated and the management of complications.

## Figures and Tables

**Table 1:** Management of blood culture-negative IE (BCNIE).

Study	Year	Main Findings
Ebato M [57]	2018	*Authors listed the main causes of BCNIE and propose a diagnostic algorithm approach: * (1) Sterilized blood culture by previous ABT: repeat blood cultures after at least 72 h of wash out (2) Slow growing microorganisms— *Candida* spp., deficient streptococci, HACEK group: prolonged blood cultures (3) Intracellular pathogens (true BCNIE)—Coxiella B., Bartonella, Mycoplasma, Legionella: serological test (4) Non infective endocarditis search for ANA ^a^, antiphospholipides antibodies, anti-Pork antibodies ^b^, malignant tumor
Fournier PE [58]	2017	*New diagnostic algorithm increases the sensitivity of diagnosis to 78% in BCNIE* vs. *64% in a previous study:* (1)To perform on blood: -Serology for Bartonella, Brucella, Legionella pneumophila, Mycoplasma pneumoniae, Coxiella B -Specific real-time PCR for *Bartonella* sp., *C. burnetii*, *E. faecalis*, *E. faecium*, *M. hominis*, *S. aureus*, *S. oralis group*, *S. gallolyticus group*, *T. whipplei* -Broad spectrum real-time PCR for fungi and prokariotes (2) To perform on valves specimens: -Culture of valve or prosthetic materials -Specific real-time PCR -Broad spectrum real-time PCR

^a^ ANA: antinuclear antibodies; ^b^ only in presence of porcine bio-prosthetic valve.

**Table 2:** Diagnostic accuracy of ^18^FDG-PET/TC in prosthetic valve IE.

Study		Sensitivity (%)	Specificity (%)	PPV (%)	NPV (%)
Pizzi [88] (2015) *Accuracy of PET/TC* versus *DC and* versus *PET/TC + DC*	DC	51.3 (34.8–67.6)	92 (74–99)	90.9 (71.9–97.5)	54.7 (46.2–63)
	PET/TC	87.2 (72.6–95.7)	92 (74–99)	82.1 (66.7–91.3)	82.1 (66.7–91.3)
	PET/TC + DC	89.7 (75.8–97.1)	88 (68.8–97.5)	92.1 (80.1–97.1)	84.6 (68.2–93.3)
De Camargo [94] (2020) *Accuracy of PET/TC in left sided prosthesis IE*		93	90	89	94
Duval [95] (2020) *Diagnostic power of PET/TC in 70 pts with PVE and 70 pts with NVE*	PVE	Definite IE	Possible IE	Rejected IE	
	*Before* vs. *After* PET/TC ± CTA	34 vs. 47	33 vs. 17	3 vs. 6	
	NVE	Definite IE	Possible IE	Rejected IE	
	*Before* vs. *After* PET/TC ± CTA	46 vs. 49	23 vs. 19	1 vs. 2	
Wahadat [96] (2019) *Role of PET/TC in 30 pts with TAVI-IE*		Definite IE	Possible IE	Rejected IE	
	*Before* PET/TC ± CTA	7/30 (23%)	22/30 (73%)	1/30 (3%)	
	*After* PET/TC ± CTA	12/30 (40%)	12/30 (40%)	6/30 (20%)	

DC, Duke criteria; PPV, positive predictive value; NPV, negative predictive value; CTA, computed tomography angiography; pts, patients; PVE, prosthetic valve endocarditis; NVE, native valve endocarditis.

## Data Availability

The data presented in this study are available in this article.

## Abbreviations

IE: infective endocarditis; MRSA, methicillin-resistant Staphylococcus aureus; TAVI, transcatheter aortic valve implantation; PVE, prosthetic valve endocarditis; NVE, native valve endocarditis; TV, tricuspid valve; CIED-IE, cardiovascular implantable electronic device-related infective endocarditis; CDR-IE, cardiac device-related infective endocarditis; BCN-IE, blood culture-negative infective endocarditis; IV, intravenous; MR, magnetic resonance; CT, computed tomography; PET/CT, positron emission tomography/computed tomography; SPECT/CT, single-photon emission computed tomography/computed tomography, CRP, C-reactive protein; TTE, transthoracic echocardiogram; TOE, transesophageal echocardiogram; CHD, congenital heart disease.
